# One Biomarker to Rule Them All?

**DOI:** 10.1016/j.jscai.2025.104006

**Published:** 2025-09-18

**Authors:** Alexander G. Truesdell, Holger Thiele

**Affiliations:** a Virginia Heart/Inova Schar Heart and Vascular, Falls Church, Virginia; b Heart Center Leipzig at Leipzig University, Leipzig, Germany

**Keywords:** Cardiogenic shock, heart failure, lactate, myocardial infarction

Decades after the development of multiple drug, device, and interventional support strategies, acute myocardial infarction and heart failure cardiogenic shock (CS) remain associated with unacceptably high early and late morbidity and mortality. Recent initiatives, most notably the Society for Cardiovascular Angiography & Interventions (SCAI) SHOCK classification and Shock Academic Research Consortium, have focused attention on better standardization of definitions for CS etiology, phenotype, and stage, while acknowledging heterogeneity and dynamic clinical trajectories.^1^^,^^2^

Since then, in recognition of the highly time-sensitive mortality of CS due to rapidly progressive tissue hypoperfusion and multiorgan system dysfunction, additional efforts have been directed toward earlier suspicion, diagnosis, and intervention for CS. A minimal core set of clinical, laboratory, and imaging data readily obtainable regardless of patient location or resource infrastructure is an unmet need, and the optimal mode of assessing (and serially reassessing) microcirculation and end-organ perfusion on top of macrocirculation also remains undetermined. All of these are important prerequisites to identifying optimal hemodynamic and perfusion targets and the preferred sequence and combination of pharmacologic, device-based, and procedural therapies for both clinical and research purposes.

The success of the SCAI SHOCK staging system lies in its thoroughness and simplicity, which make it accessible and applicable to prehospital, emergency department, cardiac catheterization laboratory, operating room, and intensive care unit staff at all levels of training and experience across different facilities and geographies. The schema is designed for both rapid initial diagnosis and repeated reassessment, and these serial reassessments have been linked to prognosis in retrospective analyses.^3^ The staging system also emphasizes important distinctions between hypotension and hypoperfusion. Data derived from physical examination (systemic and pulmonary volume assessment and cardiac and pulmonary auscultation), noninvasive hemodynamics (heart rate and blood pressure), electrocardiogram, imaging studies (chest x-ray and lung and cardiac ultrasound), laboratory tests, and more detailed invasive hemodynamic parameters (central venous pressure, systemic vascular resistance, cardiac power output, and pulmonary artery pulsatility index) are intended to be collected and reassessed at regular intervals to serially refine prognosis and management strategies. These macrovascular assessments, however, may not reliably reflect microvascular tissue perfusion, oxygenation, and end-organ function.

In this issue of *JSCAI*, Naidu et al^4^ advocate for incorporating immediate and repeated lactate testing into both CS research initiatives and routine daily care and propose “door-to-lactate clearance” (DLC) as a potential new actionable metric to aid in more rapidly identifying pre-, early-, and overt-CS, linking macrovascular hemodynamics and microvascular perfusion, tracking clinical trajectories, and driving follow-on management decisions. As highlighted by the authors, baseline and serial lactate levels correlate with mortality in multiple landmark clinical CS trials and registries, including the CardShock,^5^ Intra-Aortic Balloon Pump in Cardiogenic Shock II (IABP-SHOCK II),^6^ Culprit Lesion Only PCI vs Multivessel PCI in Cardiogenic Shock (CULPRIT-SHOCK),^7^ Dobutamine Compared to Milrinone in the Treatment of Cardiogenic Shock (DOREMI),^8^ and Danish-German Cardiogenic Shock (DanGer Shock) trials,^9^ and the National Cardiogenic Shock Initiative.^10^ These and other studies further demonstrate that elevation of lactate on presentation, persistence of lactate elevation over time, and the timing and extent of lactate clearance are all predictors of mortality in cardiac and noncardiac critical illness, making lactate an attractive biomarker for research and clinical purposes.

The authors highlight lactate as a suitable surrogate for a patient’s metabolic state and tissue-level perfusion and advocate for protocolized lactate assessment and reassessment performed at discrete frequent intervals in addition to current commonly accepted clinical and hemodynamic testing. They further highlight lactate and lactate clearance as actionable data that may inform both prognosis and clinical trajectory, trigger earlier management discussions, and inform tailored therapeutic interventions prior to the development of refractory or irreversible CS and multiorgan failure. This aligns with contemporary searches for optimal measurable macro- and microcirculatory tests and treatment targets.

While we must always beware of the “tyranny of metrics” in medicine and acknowledge that DLC does not currently merit elevation to the level of other longstanding evidence-based metrics such as door-to-balloon therapy for ST-elevation myocardial infarction, advocating for increased recognition and use of lactate measurement and serial lactate testing is still a worthy clinical and research goal.^11^ Improvements in CS survival are presently hampered by inadequate clinical suspicion, late diagnosis and initiation of clinical therapies, delayed recognition of clinical deterioration, and slow modifications of pharmacologic and device-based treatment strategies (and/or transfer to higher levels of care). This may be of greatest significance among the very large SCAI stage B (“beginning”) and normotensive SCAI C (“classic”) patient population in whom CS often goes undiagnosed and clinical deterioration unrecognized until patients devolve to more lethal (but more obvious) SCAI stages D (“deteriorating”) and E (“extremis”). Any test or intervention seeking to address this shortcoming in current CS care should be applauded (and further investigated).

Just as there is unlikely to ever be a single stand-alone drug or device to treat a complex syndrome like CS, lactate is similarly unlikely to be the one biomarker to “rule them all.”^12^ Lactate is, however, a simple, cost effective, bedside, point-of-care tool to aid CS diagnosis, serial reassessment, and clinical decision making along with physical examination, imaging, laboratory, and hemodynamic investigations. Prior to broad clinical implementation, serial lactate testing and lactate clearance timing and degree should be included in large registries and randomized clinical trials to further explore the DLC hypothesis and relationships between lactate levels and lactate clearance and specific CS therapies. In the end, early identification and fast and effective normalization of end-organ perfusion remain key goals in CS care, and the authors’ DLC call to action deserves further discussion and investigation.
